# Comprehensive Identification and Expression Profiling of the VQ Motif-Containing Gene Family in *Brassica juncea*

**DOI:** 10.3390/biology11121814

**Published:** 2022-12-14

**Authors:** Jie Zheng, Haibo Li, Ziqi Guo, Xiaoman Zhuang, Weifeng Huang, Cui Mao, Huimin Feng, Yang Zhang, Hao Wu, Yong Zhou

**Affiliations:** 1 Guangdong Provincial Key Laboratory of Utilization and Conservation of Food and Medicinal Resources in Northern Region, Shaoguan University, Shaoguan 512000, China; 2 Henry Fok College of Biology and Agriculture, Shaoguan University, Shaoguan 512000, China; 3 College of Bioscience and Bioengineering, Jiangxi Agricultural University, Nanchang 330045, China

**Keywords:** *Brassica juncea*, VQ motif-containing proteins, cold stress, expression pattern

## Abstract

**Simple Summary:**

Plant valine-glutamine (VQ) motif-containing proteins are a type of plant-specific transcription factor (TF), which contain a short and conserved amino acid motif (FxxhVQxhTG). Recent studies showed that VQ proteins play key roles in various developmental processes and abiotic/biotic stresses in plants. In this study, we identified 120 *VQ* genes in mustard (*Brassica juncea*), and their phylogenetic relationship, sequence characteristics, conserved motif, gene structure, genome distribution, gene duplication, and *cis*-element in promoters were also determined. In addition, the organ expression profiles of the *BjuVQ* genes were analyzed based on RNA-seq data, and the expression profiles of the *BjuVQ* genes under cold stress were also examined. These results provide a basis for further elucidation of the biological function of *BjuVQ* genes in mustard.

**Abstract:**

Valine-glutamine (VQ) motif-containing proteins are a class of highly conserved transcriptional regulators in plants and play key roles in plant growth, development, and response to various stresses. However, the *VQ* family genes in mustard have not yet been comprehensively identified and analyzed. In this study, a total of 120 *VQ* family genes (*BjuVQ1* to *BjuVQ120*), which were unevenly distributed on 18 chromosomes (AA_Chr01 to BB_Chr08), were characterized in mustard. A phylogenetic tree analysis revealed that the BjuVQ proteins were clustered into nine distinct groups (groups I to IX), and members in the same group shared a highly conserved motif composition. A gene structure analysis suggested that most *BjuVQ* genes were intronless. A gene duplication analysis revealed that 254 pairs of *BjuVQ* genes were segmentally duplicated and one pair was tandemly duplicated. Expression profiles obtained from RNA-seq data demonstrated that most *BjuVQ* genes have different gene expression profiles in different organs, including leaf, stem, root, flower bud, pod, and seed. In addition, over half of the *BjuVQ* genes were differentially expressed at some time points under low temperature treatment. The qRT-PCR data revealed that *BjuVQ23*, *BjuVQ55*, *BjuVQ57*, *BjuVQ67*, *BjuVQ100*, and *BjuVQ117* were upregulated in response to cold stress. Taken together, our study provides new insights into the roles of different *BjuVQ* genes in mustard and their possible roles in growth and development, as well as in response to cold stress.

## 1. Introduction

Low temperatures are one of the most important ecological factors that limit plant growth and development and consequently decrease the yield and production, by causing changes in cell physiological and biochemical statuses including cell membrane fatty acid composition, reactive oxygen species (ROS) accumulation, and gene expression level [1]. Generally, these cold stress-responsive genes can be devided into two groups, with one group encoding proteins and enzymes involved in stress tolerance and the other group encoding protein kinases and transcription factors of signal transduction in response to cold stress [2].

VQ proteins play important roles in plant response to abiotic stresses, such as salt, drought, low nitrogen, and cold stress. In banana (*Musa acuminata*), MaVQ5 represses the activity of MaWRKY26 in activating jasmonate acid (JA) biosynthetic genes in response to cold stress [3]. In sugarcane (*Saccharum spontaneum*), the expression of *SsVQ57*, *SsVQ73,* and *SsVQ76* was significantly increased after cold treatment, suggesting that *SsVQ* genes might participate in the response to cold stress [4]. In *Arabidopsis thaliana*, AtVQ9 acts as a negative regulator of salt stress response, because *AtVQ9* overexpressing lines exhibited a hypersensitive phenotype, while it was the opposite for the *vq9* mutant [5]. In apple (*Malus domestica*), MdVQ37 negatively affects the salt stress resistance by regulating reactive oxygen species elimination, ion homeostasis, and the expression of stress-related transcription factors [6]. The overexpression of *PeVQ28* from Moso bamboo (*Phyllostachys edulis*) in *A. thaliana* could positively regulate the salt stress response mediated by the ABA signaling pathway [7]. Wheat TaVQ14 could enhance the salt and drought resistance of *A. thaliana* seeds by scavenging reactive oxygen species and upregulating genes related to salt and drought stress responses [8]. After drought treatment, 22 out of the 39 *OsVQ* genes were upregulated in rice, while three were downregulated [9]. Soybean *GmVQ53* and *GmVQ58* play important roles in nitrogen metabolism and are significantly induced in both roots and shoots under low nitrogen treatment [10]. VQ proteins were also reported to regulate plant disease resistance. For example, in *A. thaliana*, AtVQ12 and AtVQ29 negatively regulate plant basal resistance against necrotrophic fungal pathogen *Botrytis cinerea* [11], while AtVQ10 plays an opposite role by interacting with AtWRKY8 [12]. In rice, OsVQ13 regulates JA-mediated resistance to the bacterial pathogen *Xanthomonas oryzae* by activating the Os–PK6–OsWRKY45 signaling pathway [13].

Valine-glutamine (VQ) proteins, which have the conserved short FxxhVQxhTG amino acid sequence motif, are a class of plant-specific proteins mostly located in the nucleus [14]. In the VQ conserved motif, F represents phenylalanine; x indicates any amino acid; h represents hydrophobic residue; V stands for valine; Q represents glutamine; T indicates tryptophan, and G represents glycine. This motif may have a significant impact on the function of VQ proteins [15]. For example, the mutation of the VQ motif would alter the interaction between VQ proteins and other partner proteins. When the residue of LVQK of the VQ motif was mutated to EDLE of *A*. *thaliana* VQ20, VQ20M could no longer interact with WRKY2 and WRKY34 in yeast and failed to rescue the phenotype in the complemented lines [16]. At the genome-wide level, the conserved domain has been used to classify the multigene family of VQ proteins, and the *VQ* gene family members have been widely identified in various plant species, such as 20 members in sunflower (*Helianthus annuus*) [17], 26 in tomato (*Solanum lycopersicum*) [18], 32 in cucumber (*Cucumis sativus*) [19], 34 in *A. thaliana* [20], 39 in rice (*Oryza sativa*) [9], 59 in tobacco (*Nicotiana tabacum*) [21], 61 in maize (*Zea mays*) [22], 74 in soybean (*Glycine max*) [10], 113 in wheat (*Triticum aestivum*) [23], and a total of 268 in *Gossypium* species [24].

In the *Brassica* species, 57 *VQ* genes in Chinese cabbage (*Brassica rapa*) [25] and 118 *VQ* genes in canola (*Brassica napus*) [26] have been identified through bioinformatics analysis of the genomes. At the adult plant stage, *BnVQ7* overexpression lines displayed enhanced resistance to the infection of *Leptosphaeria maculans*, which causes blackleg disease in canola [26]. However, the genomic information and expression patterns of *VQ* genes in mustard (*Brassica juncea*) remain unknown. In this work, *VQ* family genes were identified in mustard, and their phylogeny, gene structure, conserved motifs, chromosomal location, and gene duplications, as well as their expression levels under cold stress were examined. Our findings will provide a foundation for the future and reveal the roles of mustard *VQ* genes involved in plant stress response.

## 2. Materials and Methods

### 2.1. Plant Materials

The plant materials used in this study were *Brassica juncea*. The seeds were germinated on wet filter paper at 26 °C. The germinated seedlings were cultivated in growth chambers at 14 h/10 h (day/night) cycle at 26 °C. The 21-day-old seedlings were used for cold stress treatment.

### 2.2. Leaf Sampling and Transcriptome Analysis

The transcriptome data of mustard were obtained by collecting the mustard leaves under cold stress treatment. For the cold stress treatment, mustard plants were cultured at 4 °C for 14 h during the day and 10 h during the night with a humidity of 80% (the plants to be treated were placed in a light incubator for preculture for 72 h). During the culture, three groups of leaf samples (three biological replicates) were collected at 0 h (without cold stress treatment) and 1 h, 3 h, 6 h, 10 h, and 24 h after treatment. The total RNA of experimental samples was extracted using the TRIzol Reagent (Invitrogen, Waltham, MA, USA). A total of 3 µg RNA extracted from each sample was used to generate sequencing libraries using NEBNext^®^ UltraTM RNA Library Prep Kit (New England Biolabs, Ipswich, MA, USA), and index codes were added per each RNA sample. Complementary DNA (cDNA) fragments were obtained with a length of 250–300 bp. PCR assays were conducted using universal PCR primers, Phusion High-Fidelity DNA polymerase, and Index (X) Primer (New England Biolabs, Ipswich, MA, USA). Clustering of the index-coded samples was conducted on a cBot Cluster Generation System using TruSeq PE Cluster Kit v3-cBot-HS (Illumina, San Diego, CA, USA). The 150-bp paired end reads were sequenced on an Illumina NovaSeq platform for each library. Raw data were filtered using the NGS QC Toolkit (v2.3.3) for quality control [27]. The transcriptomic data analysis was performed with the classical HISAT2 (v2.2.1)—StringTie (v2.2.0) pipeline with default parameters [28,29]. TPM (transcripts per million) values were extracted as the expression level of the gene.

### 2.3. Identification of the VQ Gene Family in Mustard

The animo acid sequences of Arabidopsis and rice VQs were obtained from the Phytozome (https://phytozome.jgi.doe.gov/ accessed on 5 November 2022, reference genome TAIR10) and Rice Genome Annotation Project (http://rice.plantbiology.msu.edu/ accessed on 5 November 2022, reference genome MSU7) and then used as query sequences and blasted in Braju_tum_V2.0 protein database. In addition, the Hidden Markov Model (HMM) profiles of the VQ domain (Accession ID PF05678) were downloaded from the Pfam database (https://pfam.xfam.org/ accessed on 5 November 2022) and used for HMM search against the mustard genome database with hmmsearch software (v3.3) with default parameters. After manual removal of repeated sequences, the candidate sequences were submitted into the SMART (http://smart.embl-heidelberg.de/ accessed on 5 November 2022) and rechecked by HMMER (https://www.ebi.ac.uk/Tools/hmmer/ accessed on 5 November 2022) to verify the presence of the VQ motif.

### 2.4. Gene Structure, Conserved Motif, and Promoter Analysis

The sequences of the BjuVQ proteins were submitted into the Protparam tool in ExPASy (https://web.expasy.org/protparam/ accessed on 5 November 2022) to examine the biophysical properties, including protein length (aa), molecular weight (MW), and isoelectric point (*p*I). The gene information, including location of exons, introns, and untranslated region of each *BjuVQ* gene, was downloaded from BRAD (http://brassicadb.cn accessed on 5 November 2022), and the gene structure was analyzed by using TBtools [30]. The conserved motifs of the BjuVQ proteins were investigated with MEME (https://meme-suite.org/meme/tools/meme accessed on 5 November 2022) by setting the number of motifs as 10. For analysis of *cis*-elements in the promoters of the *BjuVQ* genes, the promoter sequences (2 kb upstream of the translation start site) of the *BjuVQ* genes were submitted to the PlantCARE database to analyze the hormone- and stress-responsive *cis*-elements.

### 2.5. Multiple Sequence Alignment and Phylogenetic Analysis

Multiple sequence alignment of the BjuVQ proteins was performed with Clustal Omega (https://www.ebi.ac.uk/Tools/msa/clustalo/ accessed on 5 November 2022), and the alignment results were displayed with the Geneious (v4.8.3) software. A *phylogenetic* tree of VQ proteins from mustard, *A. thaliana*, and rice was constructed with the MEGA (v7.0.26) software using the neighbor-joining (NJ) method with 1000 boostrap replicates.

### 2.6. Chromosomal Location and Duplication Analysis

The chromosomal location information of each *BjuVQ* gene was obtained from BRAD, and a chromosomal location map of all *BjuVQs* was constructed using the Gene Location Visualize of TBtools. The gene duplication events of the *BjuVQ* genes were analyzed by the WGDI (v0.6.1) software [31] and visualized by shinyCircos [32].

### 2.7. Expression Analysis of BjuVQ Genes Based on Transcriptome Data

The expression data of different organs of *B. juncea* landrace Sichuan Huangzi including leaf, stem, root, flower bud, pod, and seed were downloaded from NCBI under the accession number of PRJNA615316 (https://www.ncbi.nlm.nih.gov/bioproject/PRJNA615316 accessed on 5 November 2022). The expression data of *BjuVQ* genes under cold stress at different time points (0 h, 1 h, 3 h, 6 h, 10 h, and 24 h) were also generated using HISAT2-StringTie pipeline. The transcript abundance was calculated as TPM values. Those genes with average TPM values > 1 and present in at least one sample were identified as potentially expressed genes, and the TPM value for each gene in different organs was scaled to 0–1 and presented with heatmaps using R package ‘pheatmap’.

### 2.8. QRT-PCR Expression Analysis

Reverse transcription of one microgram of total RNA for each sample was performed using HiScript^®^ III Reverse Transcriptase (Vazyme Biotech Co., Ltd., Nanjing, China). Real-time PCR was conducted on a CFX Connect Real-Time system (BIO-RAD) using ChamQ Universal SYBR qPCR Master Mix (Vazyme Biotech Co., Ltd., Nanjing, China). The *TIP41* (tonoplastic intrinsic protein 41) gene was used as the reference, and each sample was assessed in triplicate of technical replication. All primers used are listed in Table S1.

## 3. Results

### 3.1. Identification and Characterization of VQ Family Genes in Brassica juncea

**Figure 1:**
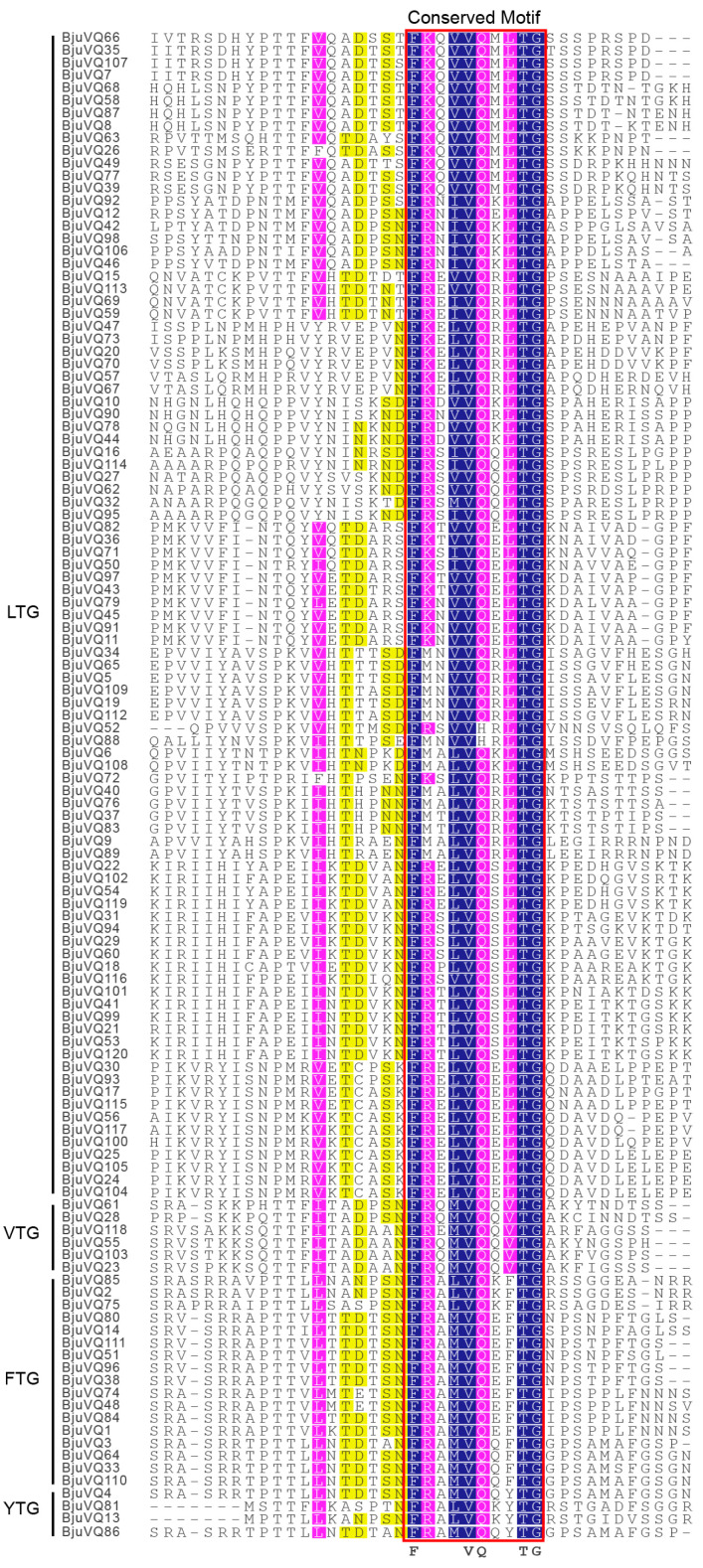
Multiple sequence alignment of the VQ domain of the 120 VQ proteins in mustard. The typical conserved domain FxxhVQxhTG is boxed in red. LTG, VTG, FTG, and YTG on the left represent four types of VQ conserved motifs in the 120 BjuVQ proteins. The color shade of the amino acid residues highlighted the homology level: dark blue = 100%, pink ≥ 80%, and yellow ≥ 60%.

The Hidden Markov Model (HMM) of the VQ motif (PF05678) was used to search and characterize the putative VQ proteins from BRAD database. A total of 120 candidate genes encoding VQ proteins were identified from the *Brassica juncea* genome, which were designated as *BjuVQ1* to *BjuVQ120* based on their location on chromosomes (Figure 1 and Table S2). Multiple sequence alignment revealed that among the 120 BjuVQ proteins, 91 had the conserved motif FxxxVQxLTG; 17 BjuVQs contained FxxxVQxFTG; six BjuVQs harbored FxxxVQxVTG, and four contained FxxxVQxYTG, whereas the core amino acid of the conserved domains of BjuVQ52 and BjuVQ88 was FxxxVHxLTG. We further analyzed the main physiological and biochemical properties of the 120 BjuVQ proteins, including the amino acid length, molecular weight (MW), theoretical isoelectric point (*p*I), and predicted grand average of hydropathy (GRAVY) values. The length of all BjuVQ proteins ranged from 91 (BjuVQ71) to 1424 (BjuVQ88) amino acids (aas), with an average of 231 aas. The MW of these BjuVQ proteins varied from 10.13 kDa (BjuVQ71) to 161.66 kDa (BjuVQ88). The *p*I value varied from 4.48 (BjuVQ82) to 10.53 (BjuVQ27), and 69 BjuVQs were basic proteins, while the remaining BjuVQs were acidic proteins. The predicted GRAVY values of all BjuVQs were negative, suggesting that all these proteins are hydrophilic. The results of the instability index analysis demonstrate that 95.83% of BjuVQs are unstable proteins, while five BjuVQs (BjuVQ13, BjuVQ18, BjuVQ81, BjuVQ94, and BjuVQ101) are stable proteins. A subcellular localization prediction showed that most BjuVQs are located in the nucleus, while four proteins (BjuVQ11, BjuVQ36, BjuVQ79, and BjuVQ97) are localized in the cytoplasm, and BjuVQ15 and BjuVQ50 are of an extracellular and a mitochondrial localization, respectively (Table S2).

### 3.2. Phylogenetic Analysis of VQ Proteins from Different Plant Species

**Figure 2:**
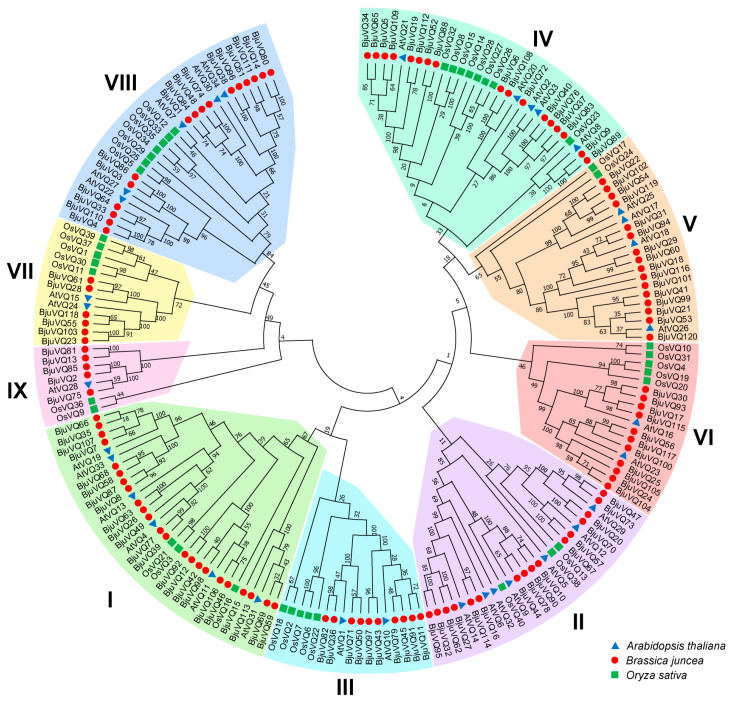
Phylogenetic tree of VQ proteins from *Brassica juncea*, *Oryza sativa*, and *Arabidopsis thaliana* using the neighbor-joining method in MEGA 7. The VQs were divided into nine groups (groups I to IX), each of which was indicated using specific color of block. The Roman numerals I–IX stand for separated groups in the phylogenetic tree. Proteins from mustard, *A. thaliana*, and rice are indicated by red circles, blue triangles, and green squares, respectively.

To explore the relationships among VQs from mustard, rice, and *A. thaliana*, we constructed an NJ phylogenetic tree using the MEGA 7.0 software (Figure 2). Based on the structural characteristics of the protein sequences, the 120 BjuVQs could be clustered into nine distinct groups (groups I to IX) according to the previous classification of VQ proteins in *A. thaliana* and rice [9]. The largest group (group I) comprised 23 BjuVQs, while the smallest group (group IX) had only five BjuVQs. An evolutionary relationship analysis showed that the VQ proteins of mustard have a closer relationship with those of *A. thaliana*, which belongs to the same *Cruciferae* family with mustard. These results suggest that the VQ proteins of mustard are closer to those of *A. thaliana* than to those of rice.

### 3.3. Conserved Motif and Gene Structural Analysis of BjuVQs

**Figure 3:**
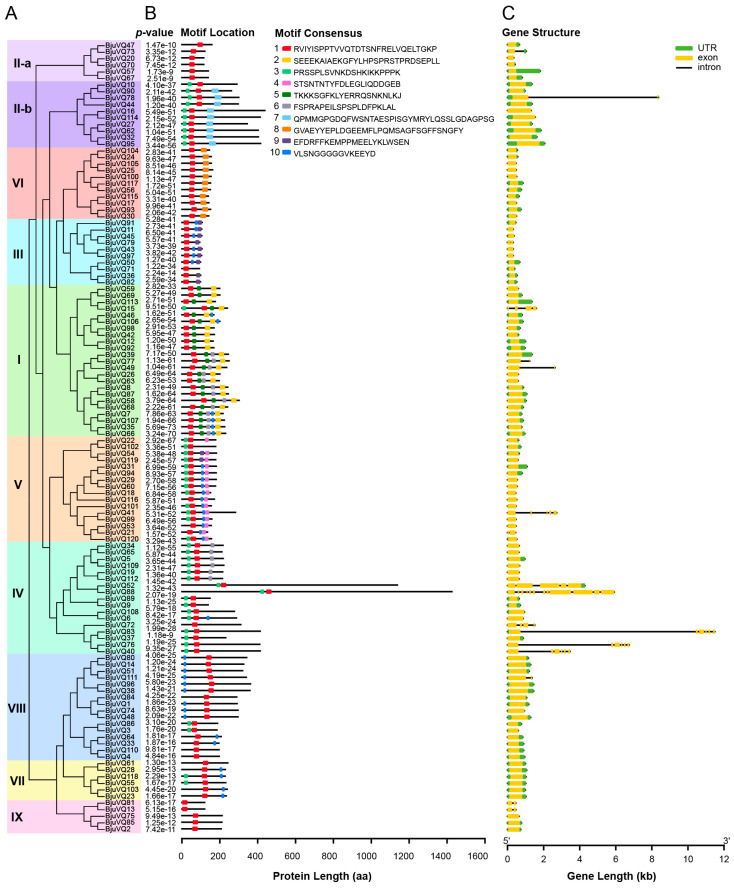
Phylogenetic analysis, conserved motifs, and gene structure of the 120 BjuVQs. (**A**) Phylogenetic tree of the 120 BjuVQs. The tree was created with 1000 bootstraps using the neighbor-joining (NJ) method in MEGA7. The Roman numerals I–IX stand for separated groups in the phylogenetic tree. (**B**) Motif composition and distribution of BjuVQs based on the phylogenetic relationship. Ten different motifs indicated by different colors were identified by MEME. The length of box and line is proportional to protein length. The *p*-value also known as “combined match *p*-value” is a probability that a motif under test would have a match to the random sequence with an equal or greater score to the largest value found in the sequence under test. (**C**) Structure of the 120 *BjuVQ* genes from the TBtools. UTRs and exons are indicted by green and yellow boxes, respectively, and introns are shown as black lines. The length of box and line is proportional to gene length.

To further elucidate the structural features of the VQ motif in BjuVQ proteins, a second phylogenetic tree was constructed in Geneious, and the conserved motif analysis of the 120 BjuVQs was conducted. A total of 10 distinct motifs were identified, among which motif 1 had a special VQ domain present in all BjuVQs (Figure 3). Combined with the phylogenetic data, it could be seen that BjuVQ proteins in the same group included a similar or identical set of motifs and structrural organization. The BjuVQs in group I had more types of motifs than those in other groups, including motif 1, motif 2, motif 3, motif 5, motif 6, and motif 10, while the BjuVQs in group II-a and group IX only contained motif 1 (Figure 3A,B). A gene structure analysis of the 120 *BjuVQs* revealed that 103 *BjuVQs* (85.83%) were intronless; nine *BjuVQs* contained one intron; three *BjuVQs* (*BjuVQ40*, *BjuVQ76*, and *BjuVQ83*) had five introns; *BjuVQ15* and *BjuVQ72* contained three introns; *BjuVQ41* and *BjuVQ52* had four introns; while *BjuVQ88* contained 11 introns (Figure 3C).

### 3.4. Genome Distribution and Gene Duplication of BjuVQ Genes

**Figure 4:**
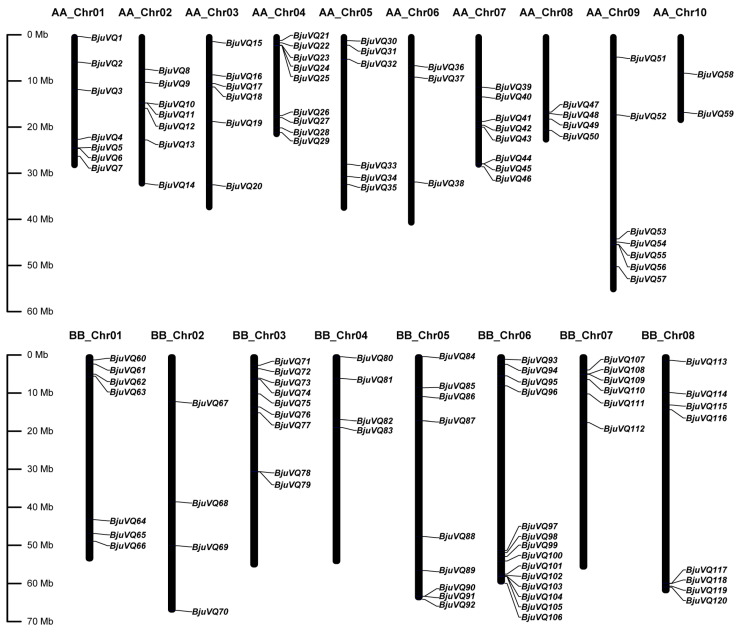
Distribution of the 120 *BjuVQ* genes in the mustard genome. The vertical black columns represent chromosomes with the gene names shown on the right. Chromosome numbers are listed above, while chromosome sizes are indicated on the left side of the figure. The length of each chromosome on the left was estimated in mega base (Mb).

**Figure 5:**
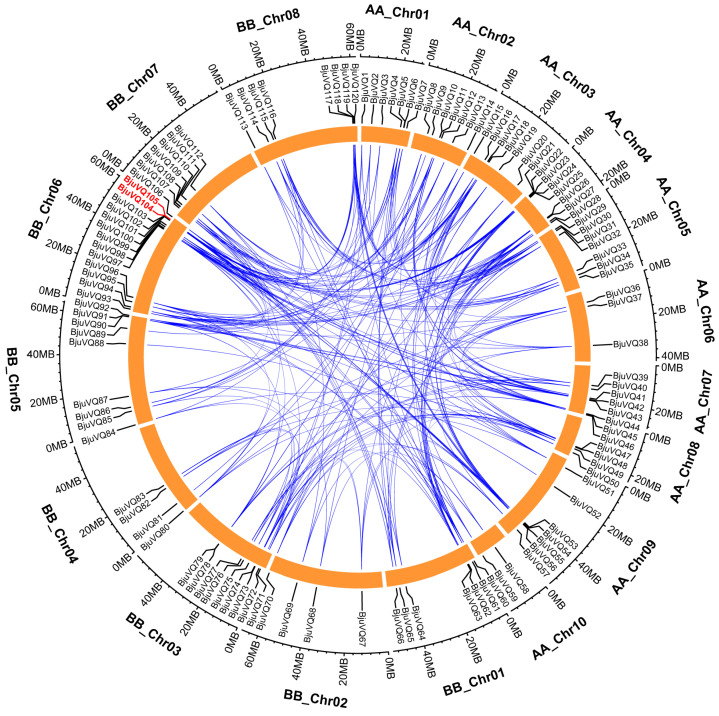
Analysis of duplication events of the 120 *BjuVQ* genes in mustard. The *Brassica juncea* chromosomes were represented by orange boxes with the gene names surrounding the boxes. Chromosome numbers are indicated at the outer edge of the circle, while the scale represents mega base (Mb). Segmental duplication gene pairs are linked by blue lines, and tandem duplication genes are shown by the red color.

To further investigate the genetic differences of *BjuVQ* genes, a chromosomal location map of 120 *BjuVQs* was constructed using the Gene Location Visualize of TBtools. The *BjuVQ* genes were unevenly distributed on 18 chromosomes, with the number of genes on each chromosome ranging from two (AA_Chr10) to 14 (BB_Chr06) (Figure 4). To explore the genomic evolution and gene family expansion, the gene duplication events of the 120 *BjuVQ* genes were determined by WGDI. As a result, 254 pairs of genes were found to be involved in segmental duplication events distributed on different chromosomes, while one pair of genes (*BjuVQ104* and *BjuVQ105*) were tandem duplication genes on chromosome BB_Chr06 (Figure 5). These results suggest that segmental duplication is predominant for *VQ* gene expansion in mustard, though it also involves certain tandem duplication.

### 3.5. Analysis of cis-Elements in the Promoters of the BjuVQ Genes

**Figure 6:**
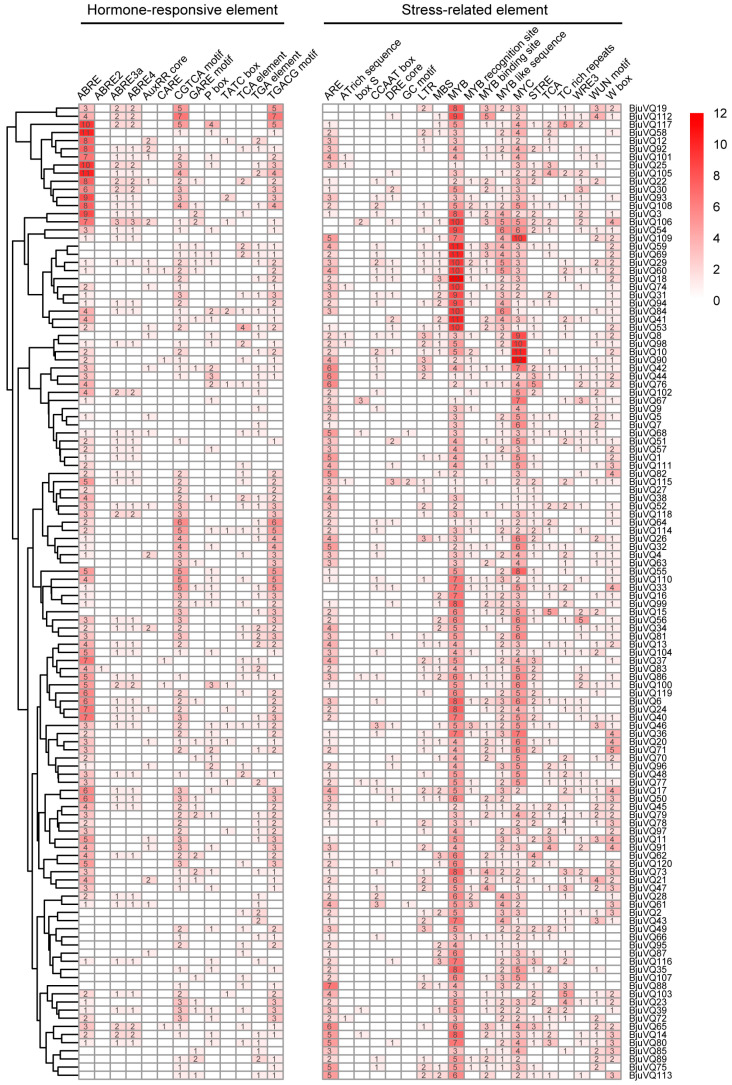
Number of each *cis*-element in the *BjuVQ* gene promoter region (2 kb upstream the translation start site). The degree of red colors represents the number of *cis*-elements upstream of the BjuVQs. A cluster dendrogram is shown on the left.

To better understand the function and regulatory mechanisms of the 120 *BjuVQs*, the *cis*-elements in their promoter regions were determined using the PlantCARE tool. A total of 32 types of *cis*-elements were found, including 13 hormone-responsive elements and 19 stress-related elements (Figure 6). The 13 hormone-responsive *cis*-acting elements comprised four ABA-responsive elements (ABRE, ABRE2, ABRE3a, and ABRE4), four gibberellin-responsive elements (CARE, GARE-motif, P-box, and TATC-box), two auxin-responsive elements (AuxRR core and TGA element), two MeJA-responsive elements (CGTCA motif and TGACG motif), and one SA-responsive element (TCA element) (Figure 6). The 19 types of stress-related elements included ARE, AT-rich sequence, box S, CCAAT box, DRE core, GC motif, LTR (low temperature response element), MBS, MYB, MYB recognition sites, MYB-binding site, MYB-like sequence, MYC, STRE (stress response element), TCA, TC-rich repeats, WRE3, WUN motif, and W-box.

Among the 13 hormone-responsive elements, ABRE (ACGTG), which is involved in ABA response, was the most abundant in the *BjuVQ* gene promoter regions, as 82.5% (99/120) of the *BjuVQ* genes had 1 to 11 ABRE elements (Figure 6). In addition, two MeJA-responsive *cis*-elements, CGTCA motif (CGTCA) and TGACG motif (TGACG), were also present in the *BjuVQ* gene promoter regions with the same proportion of 75.8% (91/120). Among the abiotic stress *cis*-elements, almost all the *BjuVQ* genes had MYB (expect for *BjuVQ102*) and MYC (expect for *BjuVQ50* and *BjuVQ103*). In addition, 44.17% (53/120) of the *BjuVQs* contained the cold-sensitive low-temperature response elements (LTR) (Figure 6).

### 3.6. Organ-Specific Expression Patterns of BjuVQ Genes

**Figure 7:**
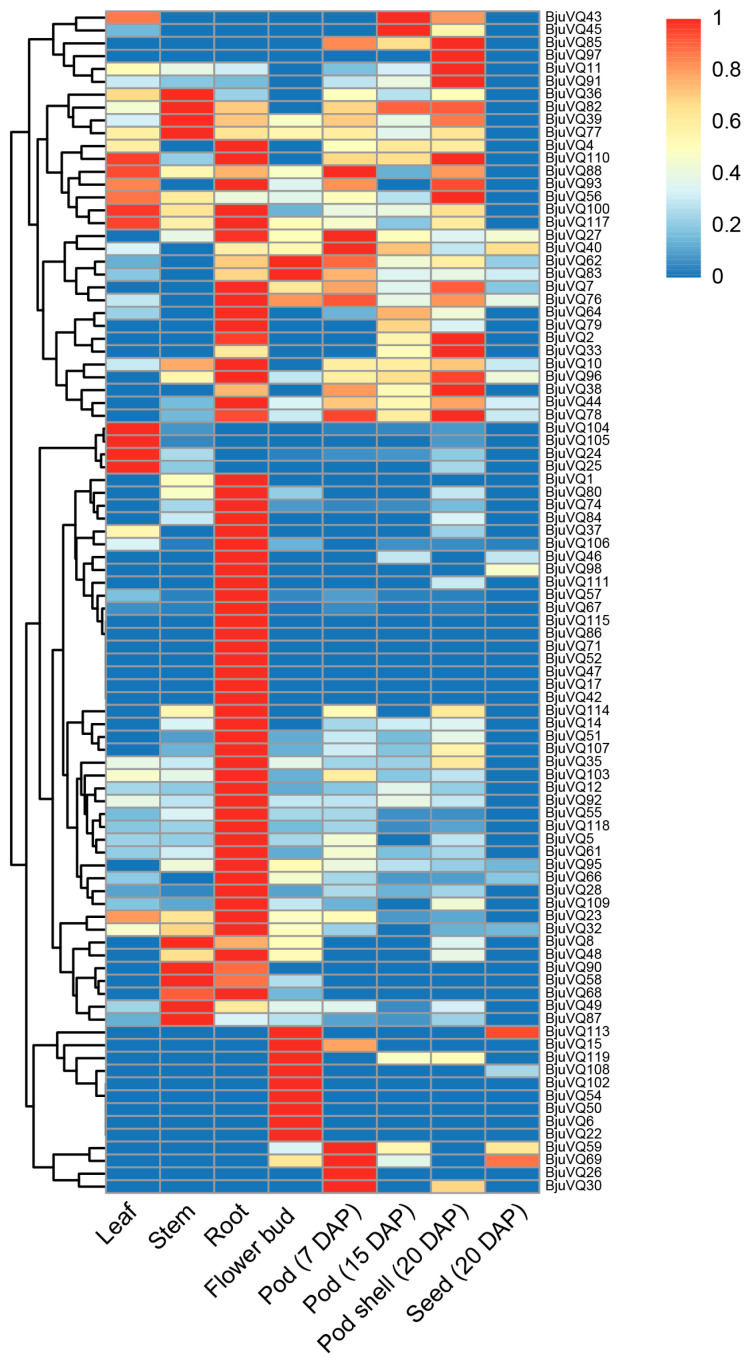
Expression profiles of *BjuVQ* genes in different organs. The organs used for the expression profiling are indicated at the bottom of each column. The TPM values of *BjuVQ* genes were calculated by RNA-seq data and normalized to 0–1. A cluster dendrogram is provided to the left of the heat map. The color gradient (red/yellow/blue) indicates the gene expression level (from high to low).

To investigate the expression patterns of the *BjuVQ* genes in mustard, the RNA-seq data in different mustard organs were obtained. A total of 92 *BjuVQ* genes showed TPM values higher than 1 in at least one of the investigated organs, and different *BjuVQ* genes exhibited a differential expression in these organs (Figure 7). Among them, most *BjuVQ* genes displayed the highest expression in roots, and some of them were found to be specifically expressed in roots. In addition, some other *BjuVQ* genes also exhibited organ-specific expression patterns. For example, *BjuVQ24*, *BjuVQ25*, *BjuVQ104*, and *BjuVQ105* were highly expressed in leaves, while *BjuVQ26*, *BjuVQ30*, *BjuVQ59*, and *BjuVQ69* showed a remarkable accumulation of transcripts in the pods at 7 DAP (Figure 7), suggesting that the *BjuVQ* genes play key roles in these organs. It should be noted that only a few *BjuVQ* genes were found to be expressed in the seeds (Figure 7), implying their possible roles in seed development. These results show that the *BjuVQ* genes participated in multiple processes during mustard growth and development.

### 3.7. Expression Patterns of BjuVQ Genes in Response to Cold Stress

**Figure 8:**
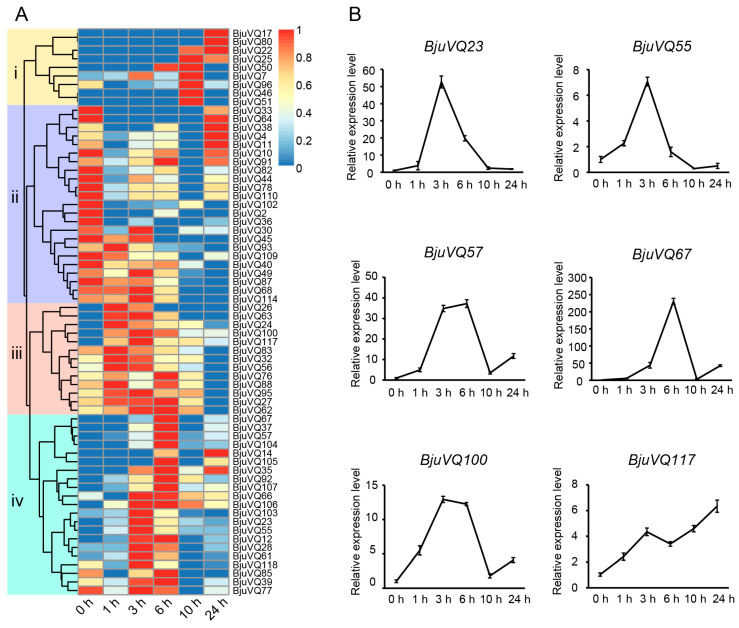
Expression levels of *BjuVQ* genes after cold treatment. (**A**) Heatmap of expression profiles of the 120 *BjuVQs* in seedlings under 4 °C treatment, and the TPM value for each gene was normalized to 0–1. A cluster dendrogram is provided to the left of the heat map, and four distinct groups (group i to iv) were colored with specific blocks. The color gradient (red/yellow/blue) of the heatmap indicates the gene expression level (from high to low). (**B**) qRT-PCR analysis of the expression profiles of six selected *BjuVQs* under cold stress.

To further study the expression patterns of *VQ* genes in mustard under low temperature, we used the 21-day-old seedlings for cold stress treatment at 4 °C. After cold stress for more than 10 h, the above-ground parts of the mustard plants started to wither. Therefore, leaf samples at 0 h (without cold stress treatment) and 1 h, 3 h, 6 h, 10 h, and 24 h after cold treatment were collected and used for RNA sequencing. Subsequently, the expression profiles of the 120 *BjuVQs* were analyzed using those transcriptome data (Table S3). In this study, 66 *BjuVQs* were identified as potentially expressed genes with the criteria of average TPM values > 1 and presence in at least one sample (Figure 8A). Among them, 23 *BjuVQs* in group ii displayed higher expression levels before cold treatment; 13 *BjuVQs* in group iii showed significant increases in transcript abundance at the earlier time points within 1 to 3 h; 21 *BjuVQs* in group iv were upregulated at 3 or 6 h, and nine *BjuVQs* in group i were upregulated at 10 or 24 h.

To determine the genes involved in cold stress response, we obtained the expression profiles of six selected *BjuVQ* genes upon exposure of the plants to a low temperature (4 °C) through qRT-PCR analysis (Figure 8B). Under cold treatment, the expression levels of three genes (*BjuVQ23*, *BjuVQ55*, and *BjuVQ100*) were rapidly upregulated and peaked at 3 h; those of *BjuVQ57* and *BjuVQ67* peaked at 6 h; while the expression of *BjuVQ117* firstly increased at 1 h and 3 h, followed by a decrease at 6 h, and then increased at 10 h and finally peaked at 24 h (Figure 8B).

## 4. Discussion

VQ proteins are a class of plant-specific proteins containing the typical conserved motif FxxhVQxhTG (x represents any amino acid, and h represents hydrophobic residues) [15]. Based on residue differences, this study identified four types of VQ conserved motifs in the 120 BjuVQ proteins, including FxxxVQxLTG (91/120), FxxxVQxFTG (17/120), FxxxVQxVTG (6/120), and FxxxVQxYTG (4/120). Previous studies have shown that there are six types of VQ motifs in *A. thaliana* (LTG, FTG, VTG, YTG, LTS, and LTD) [33], six types in Chinese cabbage (LTG, YTG, VTG, FTG, LTV, and LTS) [25], six types in tobacco (LTG, FTG, VTG, YTG, LTA, and LTV) [21], five types in wheat (LTG, FTG, ITG, VTG, and VAM) [23], five types in maize (LTG, VTG, ATG, ITG, and FTG) [22], four types in rice (ITG, LTG, VTG, and FTG) [9], four types in cucumber (LTG, FTG LTA, and VTG) [19], and three types in grapevine (LTG, FTG, and VTG) [34]. Additionally, the change of core amino acids from VQ to VH is generally regarded to be specific for monocotyledonous plants, such as rice, maize, and wheat [9,22,23]. Here, a similar case was found for the dicotyledonous plant mustard, as the core domain of BjuVQ52 and BjuVQ88 is FxxxVHxLTG.

In this study, 87.5% of *BjuVQ* genes were found to be intronless and usually encoded relatively small proteins with less than 300 amino acid residues, which is consistent with the feature of most reported *VQ* genes in plants, such as *A. thaliana* (88.2%), rice (92.5%), maize (88.5%), moso bamboo (86.2%), tobacco (83.1%), cucumber (81.25%), and wheat (91.15%) [9,19,21,22,23,33,35]. This intronless feature of genes might have resulted from the evolutionary pressure on *VQ* genes to shorten their post-transcriptional processing for a rapid response to abiotic stress [35,36]. It is worth noting that the structures of BjuVQ52 and BjuVQ88 and their encoding genes were similar and significantly different from those of other BjuVQs. For example, both proteins contained the specific core amino acids of VH as described above and had a length of 1137 and 1424 aa, respectively, which was much longer than the average value (231 aa) of BjuVQ proteins. The evolutionary relationship analysis revealed that BjuVQ52 and BjuVQ88 were closely related to each other. Moreover, these two proteins seemed to be more closely related to those in rice than to those in *A. thaliana*, which might be the reason for their unusual properties. A subcellular localization prediction indicated that about 95% of BjuVQ proteins are located in the nucleus, while four BjuVQ proteins (BjuVQ11, BjuVQ36, BjuVQ79, and BjuVQ97) are located at the cytoplasm, and two BjuVQ proteins (BjuVQ15 and BjuVQ50) are of an extracellular and mitochondrial location, respectively. According to the phylogenetic tree and conserved motif analysis, BjuVQ15 was a member of group I, while BjuVQ11, BjuVQ36, BjuVQ79, BjuVQ97, and BjuVQ50 were clustered to group III. In group I, all members were predicted to be located in the nucleus except for BjuVQ15, which might be due to its unique composition of conserved motifs relative to other members (Figure 3). The cellular location of the VQ proteins may be affected by the conserved motif of these proteins in some cases. For instance, if the conserved VVQK residues of the VQ motif are replaced by EDLE, the originally nuclear subcellular location of AtVQ9 may change to both the nucleus and cytoplasm [5]. In group III, all members of BjuVQ were more closely related to the VQ proteins of *A. thaliana* (such as AtVQ1 and AtVQ10) than to those of rice. AtVQ10 was reported to interact with WRKY8 and be exclusively localized in the nucleus [12]. In group III, BjuVQ91, BjuVQ82, BjuVQ45, BjuVQ43, and BjuVQ71, together with the most closely related members of BjuVQ11, BjuVQ36, BjuVQ79, BjuVQ97, and BjuVQ50, were all predicted to be located in the nucleus as well. Therefore, proteins not located in nucleus might be derived from their closely related nuclear-localized proteins to expand the function of the *VQ* family gene in cells.

A gene duplication analysis identified a total of 254 segmental duplication events and one tandem duplication event, indicating that segmental duplication plays a major role in the expansion of the mustard *VQ* gene family (Figure 5). These results are consistent with the findings in systematic research of gene expansion and evolution of the *VQ* gene family in 50 plant genomes, indicating that the *VQ* gene family expansion is mainly due to segmental duplication, followed by tandem duplication and mobile elements [37]. Gene duplication can result in gene functional redundancy, and the duplicated genes can develop divergent patterns in gene expression [38]. In this study, some duplicated *BjuVQ* genes possessed different expression patterns, such as *BjuVQ38/BjuVQ51*, *BjuVQ52/BjuVQ88*, and *BjuVQ69/BjuVQ113* (Figure 7). Similar findings were also observed in other plants, such as *Saccharum spontaneum* [4] and *Cucurbita pepo* [38]. Most *BjuVQ* genes show large differences in expression in some organs, such as the leaves, stems, roots, flower buds, pods, and even seeds (Figure 7), suggesting that they play various roles in the growth and development of the corresponding organs.

A phylogenetic tree analysis of the VQ proteins from mustard, rice, and *A. thaliana* clustered the 120 BjuVQ proteins into nine distinct groups (groups I to IX), and the BjuVQ proteins share a closer evolutionary relationship with the VQ proteins in *A. thaliana* (a dicot species in *Cruciferae*) than with those in monocot rice (Figure 2). In addition, members of the BjuVQ proteins in the same group with similar conserved motifs may have similar functions, indicating the evolutionary conservation of the *VQ* gene family in mustard. In this study, we determined the expression levels of three pairs of phylogenetic closely clustered *BjuVQ* genes (*BjuVQ23*/*BjuVQ55*, *BjuVQ57*/*BjuVQ67*, and *BjuVQ100*/*BjuVQ117*) under low temperature treatment (Figure 8). BjuVQ23 and BjuVQ55 were clustered into group VII with AtVQ24 (AT3G56880). BjuVQ57 and BjuVQ67 were clustered into the group II with AtVQ12 (AT2G22880), which acts as a negative regulator of plant basal resistance against *B. cinerea* [11]. BjuVQ100 and BjuVQ117 in group VI were closely related to AtVQ23, and the two *BjuVQ* genes displayed similar expression levels and were highly expressed in leaf and root (Figure 2 and Figure 7), suggesting that they may have similar functions. AtVQ23 proteins were reported to interact with WRKY33 in JA-mediated plant defense against necrotrophic pathogens or to couple with WRKY75 to regulate ABA-mediated leaf senescence [39,40].

Previous reports have shown that the expression of *VQ* genes is regulated by various abiotic stresses including low temperature [3,19,21,23,24,25]. We then investigated the possible role of *BjuVQ* genes in a cold stress response based on the transcriptome data and qRT-PCR analysis. As a result, a total of 66 *BjuVQ* genes showed obvious changes in expression at some time points under cold stress treatment (Figure 8A), and the qRT-PCR results of six selected *BjuVQ* genes were in accordance with the transcriptome data (Figure 8B). Similar results have been obtained for other plant *VQ* genes under cold stress. For example, half of the melon (*Cucumis melo*) *CmVQ* genes were significantly upregulated at one or more time points after cold treatment [41]. In *Eucalyptus grandis*, 26 out of the 27 *EgrVQ* genes were regulated under cold treatment, most of which had the highest expression at 1 h or 6 h [42]. The qRT-PCR analysis of six selected cucumber *CsVQ* genes showed that their transcription levels were remarkably altered under cold stress [19]. For the 25 selected *GmVQ* genes in soybean (*Glycine max*), the expression of 14 and 3 *GmVQ* genes was upregulated and downregulated during the cold treatment, respectively [43]. In addition, a promoter analysis indicated that the promoter regions of the *BjuVQ* genes contain many LTR *cis*-elements responsive to cold stress (Figure 6). Therefore, it can be speculated that these *VQ* genes play a vital role in cold stress response.

VQ proteins usually interact with the WRKY transcription factor to regulate various physiological processes, and WRKYs might act as binding factors to mediate the expression of both the *WRKY* and *VQ* genes to ensure an appropriate response to environmental stimuli [33]. For example, the VQ motif-containing protein IKU1 (AtVQ14) regulates endosperm growth and seed size in *A. thaliana* by interacting with AtWRKY10 [44]. Banana MaWRKY26 can physically interact with MaVQ5 to control the regulation of JA biosynthesis in response to cold stress [3]. A recent study has revealed that *A. thaliana* AtVQ23 (SIB1) and AtVQ16 (SIB2) can form a complex with WRKY75 to inhibit its function in ABA-mediated leaf senescence and seed germination [39]. The W-box motif is the binding site for the WRKY transcription factor, which is present in a vast majority of *VQ* gene promoter regions in different plants [24,43]. In this study, the W-box motif was found in the promoters of the 86 *BjuVQ* genes (Figure 6), indicating that most of these *VQ* genes may be regulated by WRKY proteins and are probably responsive to environmental stimuli [45,46].

## 5. Conclusions

In this work, a total of 120 *VQ* family genes were identified in mustard (*Brassica juncea*). A protein sequence analysis showed that there are 91 BjuVQ proteins containing LTG in the typical conserved motif FxxhVQxhTG, whereas the other BjuVQs had small variations in the conserved motif. Most *VQ* genes were found to have organ-specific expression patterns, indicating their crucial roles in different developmental processes. In addition, more than half of the *BjuVQ* genes (66/120) showed obvious changes in expression at some time points under cold stress treatment, and the qRT-PCR results of six selected *BjuVQ* genes were in accordance with the transcriptome data, suggesting that *BjuVQ* genes may also respond to cold stress in mustard. Our findings provide critical information about the further elucidation of the biological roles of *BjuVQ* genes in mustard.

## Data Availability

Not applicable.

## Supplementary Materials

The following supporting information can be downloaded at: https://www.mdpi.com/article/10.3390/biology11121814/s1, Table S1: Information of the primers used in this study; Table S2: Identification and characterization of *VQ* family genes in *Brassica juncea*; Table S3: TPM values of 120 *BjuVQ* genes under cold treatment.
